# Seroprevalence of *Angiostrongylus cantonensis* in Wild Rodents from the Canary Islands

**DOI:** 10.1371/journal.pone.0027747

**Published:** 2011-11-14

**Authors:** Aarón Martin-Alonso, Pilar Foronda, María Antonieta Quispe-Ricalde, Carlos Feliu, Basilio Valladares

**Affiliations:** 1 University Institute of Tropical Diseases and Public Health of the Canary Islands, University of La Laguna, La Laguna, Islas Canarias, España; 2 Department of Microbiology and Parasitology, University of Barcelona, Barcelona, Cataluña, España; Pennsylvania State University College of Medicine, United States of America

## Abstract

**Background:**

*Angiostrongylus cantonensis* is a lungworm of rats (Muridae) that is the causative agent of human cerebral angiostrongyliasis. The life cycle of *A. cantonensis* involves rats and mollusks as the definitive and intermediate hosts, respectively. This study was designed to increase the knowledge about the occurrence and distribution of *A. cantonensis* in its definitive host in the Canary Islands, using parasitological and serological analysis in different areas and age groups.

**Methodology/Principal Findings:**

Between 2009 and 2010, 54 black rats (*Rattus rattus*) from Tenerife were captured from six human-inhabited areas and sera samples were obtained. The lung nematodes were identified by morphological and molecular tools as *A. cantonensis*. The 31-kDa glycoprotein antigen was purified from *A. cantonensis* adult worms by electrophoresis and electroelution. Of the 54 tested rodents, 30 showed IgG antibodies against *A. cantonensis* 31-kDa antigen by ELISA. Therefore, the overall seroprevalence was 55.6% (95% CI: 42.4–68). Seroprevalent rodents were found in all the 6 areas. This 31-kDa antigen was not recognized by some sera of rats infected by other helminth species (but not *A. cantonensis*). Seroprevalence of IgG antibodies against *A. cantonensis* and prevalence based on the presence of adult worms showed significant correlation (R^2^ = 0.954, *p*<0.05).

**Conclusions/Significance:**

The present results could indicate a high prevalence of *A. cantonensis* in Tenerife and suggest the inclusion of two new zones in the distribution area of the parasite. The commonness and wide distribution of *A. cantonensis* in rats implies the presence of intermediate hosts, indicating that humans may be at risk of getting infected.

## Introduction

*Angiostrongylus cantonensis* is a lungworm of rats that has been endemic to the south Asia, Pacific Islands, Australia, and Caribbean islands. However, the global distribution of the parasite has now extended perhaps as a result of unintended importation of definitive rodent hosts on ships and aeroplanes [1]. Recently, the Canary Islands have been added to the distribution area of this nematode [2].

The life cycle of *A. cantonensis* involves rats and mollusks as definitive and intermediate hosts, respectively. Humans are accidentally infected through the consumption of raw or undercooked mollusks that contain the infective third stage larvae. Infection can also take place by eating animals that act as a paratenic host (planarians, crustaceans, frogs, monitor lizards, etc.) or ingesting contaminated fresh vegetables, including raw vegetable juice [3]. After ingestion, the nematodes are digested from tissues and enter the bloodstream in the intestine. The larvae can finally reach the central nervous system or the eye chamber, causing eosinophilic meningitis or ocular angiostrongyliasis, respectively [1].

During the past decades, several outbreaks of human angiostrongyliasis caused by *A. cantonensis* have been documented worldwide. Since 1945, more than 2800 cases of human angiostrongyliasis by *A. cantonensis* have been reported in approximately 30 countries [1]. Nowadays, this zoonosis is considered an emerging tropical disease [4]. Human cerebral angiostrongyliasis presents a broad clinical spectrum, from a mild disease to a form of eosinophilic meningitis or, uncommonly, encephalitis [5]. As a result, neurologic damage and even death may develop, especially if prompt and proper treatment is not administered [6]–[9].

The suspected diagnosis can only be confirmed upon finding and identification of *A. cantonensis* worms from the cerebrospinal fluid of infected patients, but this rarely occurs [10]–[12]. Consequently, over the past decades a great number of immunological tests have been developed to enable the diagnosis of this human angiostrongyliasis [13], [14]. These approaches include an Indirect Enzyme Linked Immunosorbent Assay (ELISA) using a 31-kDa glycoprotein from the adult worm [8], [15], [16]. This glycoprotein is among the principal antigens recognized by sera of human with *A. cantonensis* as well as sera of immunized mice, rats and rabbits [4], [15]. Previous studies in human have shown 100% diagnostic sensitivity and specificity on testing sera by ELISA, when 31-kDa glycoprotein is purified through electroelution from SDS-polyacrylamide gel [17].

The high density of rats on the Canary Islands [18] and their role in the life cycle of *A. cantonensis* highlighted the need of an epidemiological study in this archipelago. Therefore, to increase the knowledge of the occurrence and distribution of *A. cantonensis*, the first immunological screening of wild rats was performed to detect the presence of IgG against *A. cantonensis* on Tenerife (Canary Islands). Another main purpose of this work was to analyze the relationship between seroprevalence and the prevalence of adult worms of *A. cantonensis* in the different areas. Furthermore, the association between seroprevalence and several parameters that may be involved in the occurrence of this nematode was studied. The results are used to estimate the potential risk for the human population in the Canary Islands.

## Materials and Methods

### Biological samples and study area

**Figure 1:**
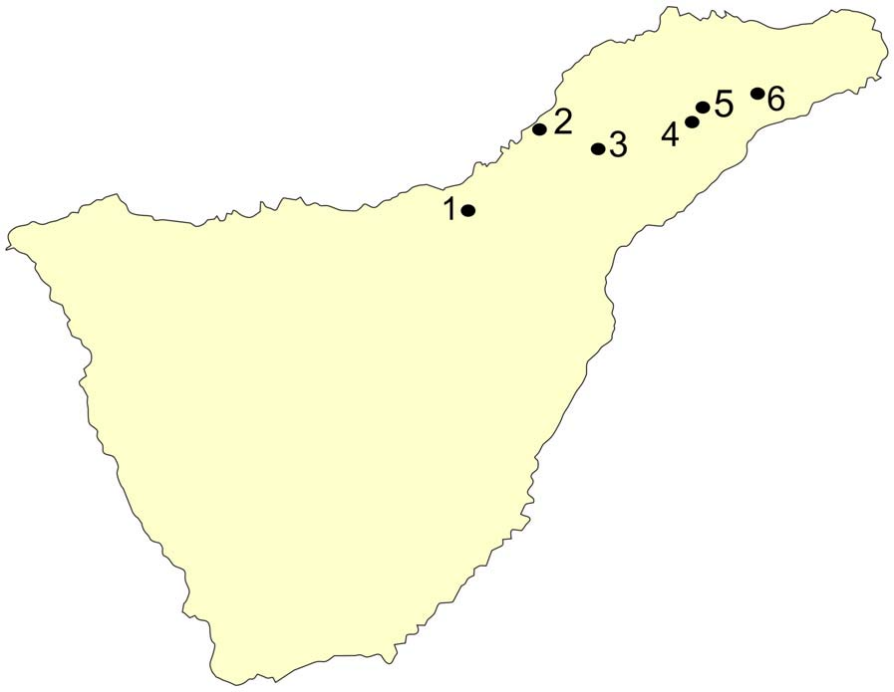
Geographical distribution of the sampling areas in Tenerife. 1, Aguamansa; 2, El Pris; 3, La Esperanza; 4, La Laguna; 5, Pedro Álvarez; 6, Pico del Inglés.

The study was carried out in the Canary Islands, an archipelago located off the northwest coast of Africa. Between 2009 and 2010, 88 black rats (*Rattus rattus*) from Tenerife (latitude 27.99 to 28.58 N; longitude 16.11 to 16.92 W) were captured. Six human-inhabited areas were analyzed (Figure 1). Rats were ranked by weight as juveniles (<100 g), adults (>130 g) [19] and subadults (100 g–130 g). Geographic region and sex of the animals sampled were also recorded. Animals were taken to the University Institute of Tropical Diseases and Public Health of the Canary Islands, euthanized with CO_2_ and bled by cardiac puncture. Blood of 54 rats was centrifuged and serum was removed and stored (in glycerol 1∶1) at −20°C until analyzed. Lesions in lungs and other tissues were recorded. The nematodes obtained from the lungs were collected, washed and conserved at −80°C until posterior molecular identification [2]. The rest of the helminths were collected and preserved in 70% ethanol until processed for morphological analysis. Cestodes and acanthocephalans were stained in aceto-ferrum carmine and mounted in Canada balsam. Nematodes were cleared in Amann lactophenol. Sera samples of rats with parasitologically proven angiostrongyliasis (three rats with *A. cantonensis* adult worms in the lungs) were obtained in order to use them as positive controls.

### Ethical statement

Animal trapping and use was approved by the Environmental Area of the Governmental competent entity the “Excmo. Cabildo Insular” of Tenerife in accordance with the Law 42/2007 and the Law 151/2001, with the expedient numbers FYF141/10 and FYF205/09.

### Parasites and parasite antigens

Adult *A. cantonensis* worms were homogenized in a small volume of phosphate buffered saline (PBS) with Complete Mini, EDTA-free protease inhibitor cocktail (Roche), using a glass tissue grinder. The suspension was then sonicated and soluble antigen was obtained as the supernatant after centrifugation at 13.000 rpm for 10 minutes. The protein content of the extract was determined using a Micro BCA™ protein assay kit (Thermo Scientific).

### Isolation of antigen from sodium dodecyl sulphate-polyacrylamide gel by electroelution

The 31-kDa antigen isolation was carried out by SDS-PAGE, according to the method of Laemmli [20], as previously described [17]. Crude antigenic extract of *A. cantonensis* (90 µg of proteins per lane) was separated on a 12% reducing SDS-polyacrylamide gel. After electrophoresis, the resolved polypeptide bands were revealed by staining with Coomassie brilliant blue R 250 (Sigma-Aldrich). Individual slots in the same gel were used to electrophorese the high and low molecular weight standards, SDS-6H and SDS-7 (Sigma-Aldrich), respectively. Once the electrophoresis was carried out, strips with the molecular standards were cut and rapidly stained with Coomassie brilliant blue R 250 to determine the region where the 31-kDa antigen of interest would be according to approximate molecular weight. A region that included the 31-kDa targeted antigen was cut into strip and the gel pieces were electroeluted using a dialysis membrane at a molecular weight cut off of 12,000–15,000 Da. Electroelution was done at 10 mA current for 12 hours at 4°C.

### Enzyme-linked immunosorbent assay (ELISA)

The *Angiostrongylus cantonensis*-specific IgG antibodies were tested by ELISA, as previously described [17]. Eluted 31-kDa antigen fraction of *A. cantonensis* at a concentration of 1 µg/ml in phosphate buffer saline was incubated in wells of microtiter plate (Thermo Scientific). Antigen was incubated 2 hours at 37°C and then overnight at 4°C. Sera and the horseradish peroxidase-conjugated goat anti-rat immunoglobulins (Sigma-Aldrich) were used at 1∶100 and 1∶5,000 dilutions, respectively, and both were incubated for 2 hours at 37°C. Binding was colorimetrically visualized following the addition of OPD (Sigma-Aldrich) for 30 minutes in the dark at room temperature. The enzymatic reaction was stopped with 200 µl of 2.5 N sulphuric acid and the plate was read spectrophotometrically at 490 nm with an ELISA reader (Bio-Rad). Final volumes of 100 µl per well were used for antigen, sera, conjugate and substrate. For each test, three negative and three positive controls were included. The negative control group of sera was obtained of both six captured rats that were negative for any parasitic infection and three Sprague-Dawley rats. The cut-off value was determined in accordance with this group.

### Statistical Analysis

Results were analyzed with SPSS 15.0 software package. Seroprevalence of IgG antibodies against *A. cantonensis* was expressed as the ratio of positive rats to the total number tested (%). Chi-square test was used to analyze the anti-*A. cantonensis* IgG seroprevalence in respect of gender and age. 95% confidence intervals (CI) for parasitologically proven prevalence and seroprevalence were calculated by Wilson method. To compare the mean optical densities among the studied areas, the Kruskal Wallis test (KW) and the Mann-Whitney (MW) post-test for single comparisons were used. The differences were considered to be statistically significant when the *p*-value was less than 0.05. Linear correlation analysis was used to assess the association between the prevalence based on the presence of adult worms and seroprevalence of *A. cantonensis* in each area.

## Results

The helminths found in rats were morphologically identified and results are shown in Table S1. In the case of the nematodes recovered from the lungs, the morphological and molecular identification was carried out as previously described [2]. Fourteen rats were positive for *A. cantonensis*. The prevalence of this parasite according to each area is summarized in Table 1.

**Table 1:** Prevalence based on the presence of *Angiostrongylus cantonensis* (P) and seroprevalence of IgG antibodies against *A. cantonensis* (SP), in *Rattus rattus* from Tenerife.

AREA	P (%) (+/n) [95% CI]	SP (%) (+/n) [95% CI]
La Esperanza	6.7 (1/15) [1.2–29.8]	33.3 (4/12) [13.8–60.9]
El Pris	42.9 (3/7) [15.8–75	83.3 (5/6) [43.7–97]
La Laguna	6.7 (1/15) [1.2–29.8]	44.4 (4/9) [18.9–73.3]
Pedro Álvarez	21.4 (9/42)[11.7–35.9]	58.8 (10/17) [36–78.4]
Pico del Inglés	0 (0/4) [0–49]	100 (4/4) [51–100]
Aguamansa	0 (0/5) [0–43.5]	50 (3/6) [18.8–81.2]
TOTAL	15.9 (14/88) [9.7–25]	55.6 (30/54) [42.4–68]

(+: positive samples; n: sample size; CI: Confidence Interval).

**Figure 2:**
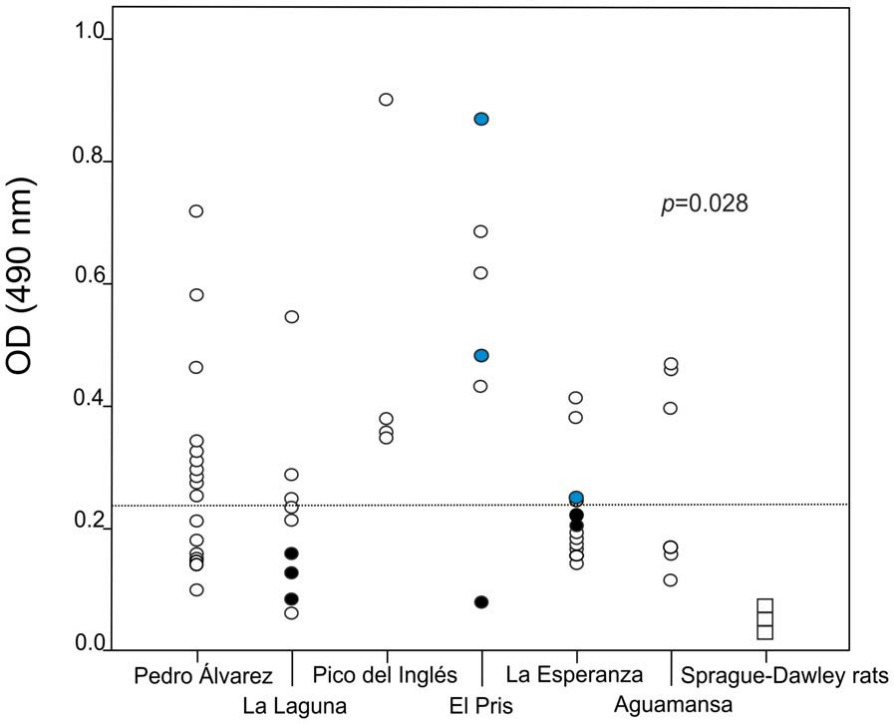
Distribution of optical density (OD) values in ELISA for detection of antibodies against *Angiostrongylus cantonensis*. The sera were divided based on the area where *R. rattus* were trapped, and the negative controls (Sprague-Dawley rats). Significant differences in mean OD values among the studied areas are given as *p*-value. (Square shape points: sera of Sprague-Dawley rats; Blue circles: sera of rats with *A. cantonensis*; Black circles: rats negative for any parasitic infection and considered as negative controls; Dotted line: cut-off value (OD = 0.235)).

The 31-kDa glycoprotein was successfully isolated from the crude worm extract of *A. cantonensis*. The OD at 490 nm (OD 490) indicated the amount of specific IgG antibodies in the sera and are shown in the figure 2. The mean OD value (X±SD) of the negative control group was 0.132±0.052. The mean plus two standard deviation OD value of the negative control group was taken as the cut-off value, considering OD>0.235 as positive results. Of the 54 tested rodents, 30 showed IgG antibodies against *A. cantonensis* 31-kDa antigen. Therefore, the overall seroprevalence was 55.6% (95% CI: 42.4–68). All the sera of the negative control group (n = 9) were found negative, while rats with parasitologically proven angiostrongyliasis showed positive results (Figure 2). Also, all the rats that had lung lesions showed positive results. The seroprevalences obtained in each area are shown in Table 1.

High levels of *A. cantonensis* infection were found early in *R. rattus*, with a seroprevalence of 33.3% among juvenile rodents. The seroprevalence increased to 60% among adult rodents, but the observed difference between age groups was not statistically significant. No difference in the seroprevalence was observed according to gender.

*A. cantonensis* occurred in all the six areas (Figure 2), but there was a significant difference in the seroprevalence of the parasite among the studied areas (KW = 12.557; *p*<0.05). The highest seroprevalence of *A. cantonensis* was found in Pico del Inglés, and it was significantly higher than those observed in La Esperanza (Mann-Whitney U, z = 2.55, *p*<0.05), La Laguna (Mann-Whitney U, z = 2.31, *p*<0.05) and Pedro Álvarez (Mann-Whitney U, z = 2.3, *p*<0.05) (table 1), all of them being humid areas. On the other hand, the mean OD value obtained in El Pris, the second most seroprevalent place, was higher than that found in La Esperanza (Mann-Whitney U, z = 2.25, *p*<0.05).

A positive linear correlation was observed between the helminthologically proven prevalence of the parasite and the seroprevalence of IgG antibodies against *A. cantonensis* (R^2^ = 0.954, slope = 1.224, 95% CI: 0.408–2.041, *p*<0.05).

In contrast, the 31-kDa antigen was not recognized by some sera of rats infected by other nematode species, such as *Calodium hepaticum*, *Syphacia muris* and *Mastophorus muris*. Negative results were also obtained for some rats infected by various cestode species, including *Taenia taeniaeformis* (metacestode) and *Hymenolepis* sp.

## Discussion

The present study represents the first immunological screening of antibodies against *A. cantonensis* in wild animals. More than half of the studied rodents were seropositive, suggesting a high prevalence of this nematode on Tenerife. In fact, the seroprevalence of *A. cantonensis* in *R. rattus* was higher than the prevalence observed by parasitological analysis previously on Tenerife [2], and in different endemic areas as Antilles (23.4% in *R. rattus*) [21], Taiwan (16.3% in *R. rattus*) [22], China (16.6% in *R. norvegicus*) [23] and Jamaica (12.9% in *R. rattus*) [24]. However, this difference could be due to the intrinsic differences between these diagnostic approaches, since not only the presence of the parasite can be detected by this immunological assay, but also past contacts with it. The high seroprevalence of IgG antibodies against *A. cantonensis* observed in our study could be explained by the fact that introduced rats in Tenerife are known to predate upon snails [25] that have been described previously as intermediate hosts of *A. cantonensis*, such as *Plutonia* sp. and *Hemicycla* sp. [3]. Furthermore, our study suggests the presence of *A. cantonensis* in two new areas, Aguamansa and Pico del Inglés, increasing our knowledge about the real distribution of this parasite in the Canary Islands.

However, these results need to be interpreted with caution. Although Eamsobhana et al. [17] have reported 100% diagnostic specificity and sensitivity on testing human sera with 31-kDa glycoprotein by ELISA, it is not necessarily the same in rats. The presence of a linear correlation between seroprevalence of IgG antibodies against *A. cantonensis* and helminthological prevalence of *A. cantonensis* in the different areas could indicate a high level of sensitivity of the 31-kDa glycoprotein on testing rats sera by ELISA. Therefore, and considering the fact that 31-kDa glycoprotein is among the principal antigens recognized by rats [4], [15], experimental studies should be carried out to determine the sensitivity and specificity of this glycoprotein for rats.

On the other hand, the discrepancy between the prevalence based on the presence of adult worms and the seroprevalence observed in this study could be explained by the presence of antibodies due to a past infection and due to the reason that not all the rats that acquire the larvae develop the adult form of the parasite. It is also possible that rodents produce antibodies against this nematode before the parasite has reached the lungs.

*Rattus rattus* and *R. norvegicus* are the main definitive hosts for *A. cantonensis* [12], [26]. This fact emphasizes importance of our study from the public health point of view, due to the high density and wide distribution of these rodent species on the Canary Islands [18]. Furthermore, most of the sampled locations of this study are inhabited by humans. For this reason, serologic tests need to be carried out to confirm infections in the human population of the Canary Islands.

The commonness and wide distribution of *A. cantonensis* in Tenerife also indicates the existence of suitable intermediate hosts of *A. cantonensis* (snails and/or slugs), the main source of human infection. In contrast to large parts of Europe, the Canary Islands are very rich in gastropod species, among which many are endemic [27]. Consequently, further research is needed to study the intermediate host range of the parasite in the Canary Islands. Although raw snails are not a part of the Canary diet, the health risk can only be evaluated with a full understanding of the intermediate host range of *A. cantonensis* and their biology.

Because this study revealed a common and widespread occurrence of the parasite in rats in Tenerife, *A. cantonensis* infection should be considered in the differential diagnosis of cases of eosinophilic meningitis. The ELISA-based method for the detection of *A. cantonensis* described in the present study allows a large-scale assessment of the disease in humans and seroepidemiological investigations, given its high specificity and sensitivity. For prevention of human infection in the Canary Islands, rodent control measures are recommended.

## Supporting Information

Table S1 **A detailed overview of the helminth species obtained from each specimen and the results of the immunological assay. (OD values at 490 nm).**
(PDF)
