# Supplementary material for: Seroprevalence of Angiostrongylus cantonensis in Wild Rodents from the Canary Islands
Source: PLoS One. 2011 Nov 14;6(11):e27747. doi: 10.1371/journal.pone.0027747 (PMC3215735; doi:10.1371/journal.pone.0027747)
Supplement: Table S1 — A detailed overview of the helminth species obtained from each specimen and the results of the immunological assay. (OD values at 490 nm). (PDF) [file pone.0027747.s001.pdf]

Supporting information.

Table S1. A detailed overview of the helminth species obtained from each specimen and the results of the immunological assay. (OD values at 490 nm).

Area	Helminth species	OD value
Pedro Álvarez	*Calodium hepaticum*	0.2995
Pedro Álvarez	*Mastophorus muris, Calodium hepaticum, Taenia taeniaeformis* , Acanthocephalan	0.3405
Pedro Álvarez	*Mastophorus muris, Calodium hepaticum, Taenia taeniaeformis*	0.1565
Pedro Álvarez	*Mastophorus muris, Calodium hepaticum,* Trematoda	0.181
Pedro Álvarez	*Brachylaima* sp., *Mastophorus muris,* Acanthocephala	0.2715
Pedro Álvarez	*Brachylaima* sp., *Calodium hepaticum, Aspiculluris* sp.	0.149
La Laguna	*Syphacia muris*	0.2345
Pedro Álvarez	*Calodium hepaticum*	0.178
La Laguna	*Trichuris muris*	0.2475
La Laguna		0.06
Pedro Álvarez	*Brachylaima* sp., *Syphacia muris*	0.3135
Pedro Álvarez	*Calodium hepaticum*	0.0985
La Laguna	*Mastophorus muris*	0.2875
La Laguna	*Trichuris muris*	0.5465
Pedro Álvarez	*Mastophorus muris, Calodium hepaticum, Taenia taeniaeformis* , *Heterakis spumosa*	0.253
Pedro Álvarez		0.3415
La Laguna		0.1575
La Laguna		0.1265
La Laguna		0.0835

Pedro Álvarez	*Mastophorus muris, Calodium hepaticum*	0.4635
Pedro Álvarez	*Calodium hepaticum, Taenia taeniaeformis*	0.2875
Pedro Álvarez	*Brachylaima* sp., *Mastophorus muris, Calodium hepaticum,* Acanthocephala	0.3255
Pedro Álvarez	*Mastophorus muris, Calodium hepaticum*	0.5825
Pedro Álvarez	*Calodium hepaticum, Brachylaima* sp.	0.7185
La Laguna	*Calodium hepaticum*	0.215
Pedro Álvarez	*Mastophorus muris, Calodium hepaticum, Taenia taeniaeformis*	0.2125
Pico del Inglés	*Mastophorus muris, Calodium hepaticum* *	0.3575
Pico del Inglés	*Mastophorus muris, Calodium hepaticum*	0.3485
Pico del Inglés	*Calodium hepaticum*	0.8995
Pico del Inglés	*Calodium hepaticum*	0.3785
El Pris		0.0785
El Pris	*Mastophorus muris, Hymenolepis* sp., *Streptopharagus* sp.	0.6185
El Pris	*Mastophorus muris, Hymenolepis* sp., *Streptopharagus* sp.	0.4325
El Pris	*Hymenolepis* sp., ***Angiostrongylus cantonensis***	0.4825
El Pris	*Mastophorus muris,* ***Angiostrongylus cantonensis***	0.8705
El Pris	*Mastophorus muris, Hymenolepis* sp.	0.6855
La Esperanza		0.4125
La Esperanza		0.3805
La Esperanza	*Mastophorus muris*	0.2065
La Esperanza		0.2225

La Esperanza	*Hymenolepis* sp.	0.1975
La Esperanza		0.1645
La Esperanza		0.221
La Esperanza		0.2515
La Esperanza		0.1965
La Esperanza	*Mastophorus muris*	0.177
La Esperanza	***Angiostrongylus cantonensis***, *Mastophorus muris*, *Syphacia muris*	0.2535
La Esperanza	*Mastophorus muris*	0.145
Aguamansa	*Mastophorus muris* *	0.4685
Aguamansa	*Aonchotheca* sp., *Hymenolepis* sp.	0.395
Aguamansa		0.1575
Aguamansa	*Aonchotheca* sp., *Hymenolepis* sp.	0.4605
Aguamansa		0.17
Aguamansa		0.116

* Lung lesions
